# Neurodegeneration and Motor Dysfunction in Mice Lacking Cytosolic and Mitochondrial Aldehyde Dehydrogenases: Implications for Parkinson's Disease

**DOI:** 10.1371/journal.pone.0031522

**Published:** 2012-02-22

**Authors:** Margaret Chia-Ying Wey, Elizabeth Fernandez, Paul Anthony Martinez, Patricia Sullivan, David S. Goldstein, Randy Strong

**Affiliations:** 1 Department of Pharmacology, University of Texas Health Science Center at San Antonio, San Antonio, Texas, United States of America; 2 Sam and Ann Barshop Institute for Longevity and Aging Studies, University of Texas Health Science Center at San Antonio, San Antonio, Texas, United States of America; 3 Geriatric Research, Education and Clinical Center, South Texas Veterans Health Care Network, San Antonio, Texas, United States of America; 4 Clinical Neurocardiology Section, Clinical Neurosciences Program, Division of Intramural Research, National Institute of Neurological Disorders and Stroke, Bethesda, Maryland, United States of America; National Institutes of Health, United States of America

## Abstract

Previous studies have reported elevated levels of biogenic aldehydes in the brains of patients with Parkinson's disease (PD). In the brain, aldehydes are primarily detoxified by aldehyde dehydrogenases (ALDH). Reduced *ALDH1* expression in surviving midbrain dopamine neurons has been reported in brains of patients who died with PD. In addition, impaired complex I activity, which is well documented in PD, reduces the availability of the NAD^+^ co-factor required by multiple ALDH isoforms to catalyze the removal of biogenic aldehydes. We hypothesized that chronically decreased function of multiple aldehyde dehydrogenases consequent to exposure to environmental toxins and/or reduced *ALDH* expression, plays an important role in the pathophysiology of PD. To address this hypothesis, we generated mice null for *Aldh1a1* and *Aldh2*, the two isoforms known to be expressed in substantia nigra dopamine neurons. *Aldh1a1^−/−^×Aldh2^−/−^* mice exhibited age-dependent deficits in motor performance assessed by gait analysis and by performance on an accelerating rotarod. Intraperitoneal administration of L-DOPA plus benserazide alleviated the deficits in motor performance. We observed a significant loss of neurons immunoreactive for tyrosine hydroxylase (TH) in the substantia nigra and a reduction of dopamine and metabolites in the striatum of *Aldh1a1^−/−^×Aldh2^−/−^* mice. We also observed significant increases in biogenic aldehydes reported to be neurotoxic, including 4-hydroxynonenal (4-HNE) and the aldehyde intermediate of dopamine metabolism, 3,4-dihydroxyphenylacetaldehyde (DOPAL). These results support the hypothesis that impaired detoxification of biogenic aldehydes may be important in the pathophysiology of PD and suggest that *Aldh1a1^−/−^×Aldh2^−/−^* mice may be a useful animal model of PD.

## Introduction

Parkinson's disease (PD) is the 2^nd^ most prevalent neurodegenerative disease. It is classically diagnosed by the presence of movement and gait abnormalities, but also involves non-motor features, including autonomic and cognitive manifestations [1]. Pathological hallmarks of PD include age-progressive degeneration of mesencephalic dopamine neurons and, in surviving neurons, the presence of Lewy bodies, cytoplasmic inclusions of which α-synuclein is a major constituent [2]. Symptoms of PD are associated with loss of dopamine in the substantia nigra and striatum [3].

Mechanisms of PD pathogenesis remain to be elucidated. Numerous studies have implicated mitochondrial dysfunction and elevated oxidative stress [4]. In addition to genetic and environmental factors that can contribute to oxidative stress and mitochondrial dysfunction, excess biogenic aldehydes may also play an important role [5]. Biogenic aldehydes are cytotoxic, can cause protein cross-linking [6] and promote α-synuclein oligomerization [7]–[9]. Elevated levels of 4-hydroxynonenal (4-HNE), the end product of lipid peroxidation, have been reported in affected brain regions of patients dying with PD and increased ratio of 3,4-dihydroxyphenlyacetaldehdye (DOPAL), the first product of dopamine metabolism by monoamine oxidase (MAO), relative to dopamine have been reported in the brains of PD patients [7], [10], [11].

Aldehyde dehydrogenases play an important role in detoxifying aldehydes in the brain. There are 19 human aldehyde dehydrogenase isoforms and 20 mouse isoforms with wide tissue distribution and localization in all subcellular compartments, including cytosol, mitochondria, endoplasmic reticulum, and nucleus [12]. In the brain, Aldh1a1 (ALDH1 in humans), a cytosolic enzyme, is highly and exclusively expressed in midbrain dopaminergic neurons [13]–[15]. *ALDH1* mRNA expression is reportedly decreased in surviving neurons of PD patients [14], [15]. Mitochondrial Aldh2, (ALDH2 in humans) is also expressed in midbrain dopamine neurons [13], [14]. In humans, a common loss of function genetic polymorphism (ALDH2*2) frequently found in northeast Asians, has been identified as a risk factor for Alzheimer's disease [16]–[18]. In addition, impaired complex I activity, a well-documented finding in PD, reduces the availability of the NAD^+^ co-factor required by multiple ALDH isoforms to catalyze the removal of biogenic aldehydes. We hypothesized that chronically decreased function of multiple aldehyde dehydrogenases consequent to exposure to environmental toxins and/or reduced *ALDH* expression, plays an important role in the pathophysiology of PD. To test this hypothesis, we created micewith double homozygous mutations in *Aldh1a1* and mitochondrial *Aldh2*, the two isozymes known to be expressed in midbrain dopamine neurons [13], [14]. Loss of these two enzymes resulted in decline in locomotor function, loss of tyrosine hydroxylase (TH) immunoreactive neurons in the substantia nigra pars compacta, and reductions in monoamines and metabolites in the neostriatum.

## Results

### Effect of Genotype and Age on Grip Strength and Body Weight

**Figure 1:**
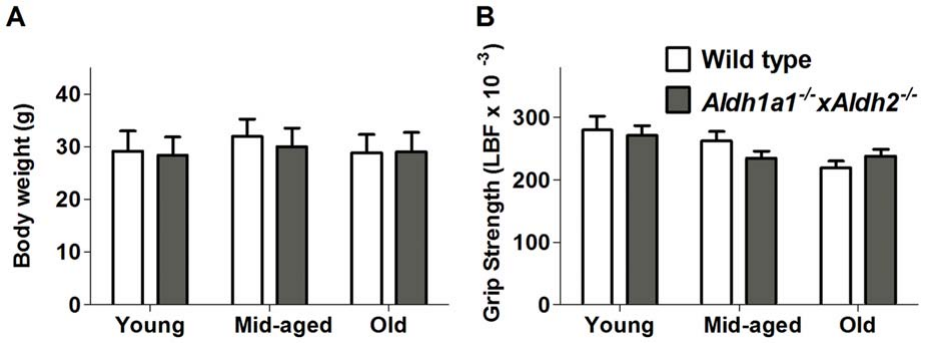
Effect of genotype and age on body weight and muscle strength. (A) Body weight and (B) grip strength. Data are expressed as the mean ± SEM of the following number of mice of each age and genotype: young (wt = 34, ko = 35), middle-aged (wt = 23, ko = 46) and old (wt = 25, ko = 29) male mice.

Figure 1A shows that there was no effect of genotype on body weight (F_1,176_ = 2.605, *p* = 0.108). However, there was a significant effect of age on body weight (F_2,176_ = 6.527, *p* = 0.002). There was no interaction between genotype and age on body weight (F_2,176_ = 1.213, *p* = 0.30) Similarly in Figure 1B, there was no effect of genotype on grip strength (F_1,103_ = 0.0357, *p* = 0.850) in *Aldh1a1^−/−^×Aldh2^−/−^* mice, but there was a significant effect of age on grip strength (F_2,103_ = 5.558, *p* = 0.015). There was no interaction between genotype and age on grip strength (F_2,103_ = 1.812, *p* = 0.168). There were also no differences in tests of general locomotor activities measured in an open field, a light/dark open field activity test and in 24-hour activity tests (Figure S1).

### Effect of Genotype and Age on Rotarod Performance

**Figure 2:**
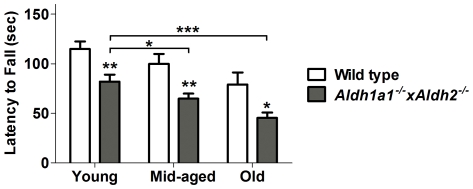
Effect of genotype and age on rotarod performance. Data are expressed as the mean ± SEM of measures from the following number of mice of each age and genotype: young (wt = 34, ko = 35), middle-aged (wt = 23, ko = 46) and old (wt = 25, ko = 29) male mice. Asterisks above bars indicate significant difference between age-matched wt and ko groups. Asterisks above lines indicate significant difference between indicated means. *P<0.05; **P<0.01; ***P<0.001.

Figure 2 shows the results of measuring the effects of genotype and age on motor performance on the accelerating rotarod. There was a significant effect of age (F_2,189_ = 13.301, *p*<0.001) and genotype (F_1,189_ = 29.351, *p*<0.001) on rotarod performance. There was no statistically significant interaction between age and genotype (F_2,189_ = 0.018, *p* = 0.983). Student-Newman-Kuels *post-hoc* comparison of individual means revealed significant differences at each age between *Aldh1a1^−/−^×Aldh2^−/−^* mice and their age-matched wild type controls. Thus, the *Aldh1a1^−/−^×Aldh2^−/−^* mice spent significantly less time on the rotarod than age-matched wild type mice with a mean percentage reduction of 29% in the young mice, 35% reduction in middle-aged mice and a 43% decline in the mean latency to fall in the old mice as compared to wild type mice of the same age.

### Effect of Genotype and Age on Gait Performance

**Figure 3:**
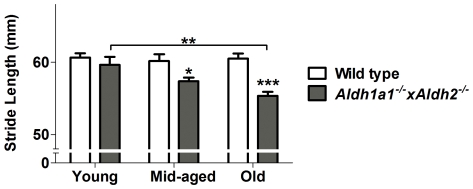
Effect of genotype and age on gait. Data are expressed as mean ± SEM of the following number of each age and genotype: young (wt = 8, ko = 9), middle-aged (wt = 11, ko = 14) and old (wt = 18, ko = 23) male mice. Asterisks above bars indicate significant difference between age-matched wt and ko groups. Asterisks above lines indicate significant difference between indicated means. *P<0.05; **P<0.01; ***P<0.001.

We then analyzed motor function by gait analysis in a subset of animals. As shown in Figure 3, automated treadmill gait analysis revealed a significant effect of age (F_2,77_ = 7.26, *p* = 0.018) and genotype (F_1,77_ = 19.76, *p*<0.0001) on stride length. *Post-hoc* analysis revealed that stride length was significantly reduced in middle-aged and old *Aldh1a1^−/−^×Aldh2^−/−^* mice as compared to their age-matched wild-type controls. Interaction accounts for approximately 6.57% of the total variance (F_2,77_ = 3.81, *p* = 0.026), suggesting that stride length might change with age differently in wild type and *Aldh*-deficient animals. No significant effects of age and genotype were observed on stride frequency and stride duration.

### L-DOPA Alleviated Motor Deficits in *Aldh1a1^−/−^×Aldh2^−/−^* Mice

**Figure 4:**
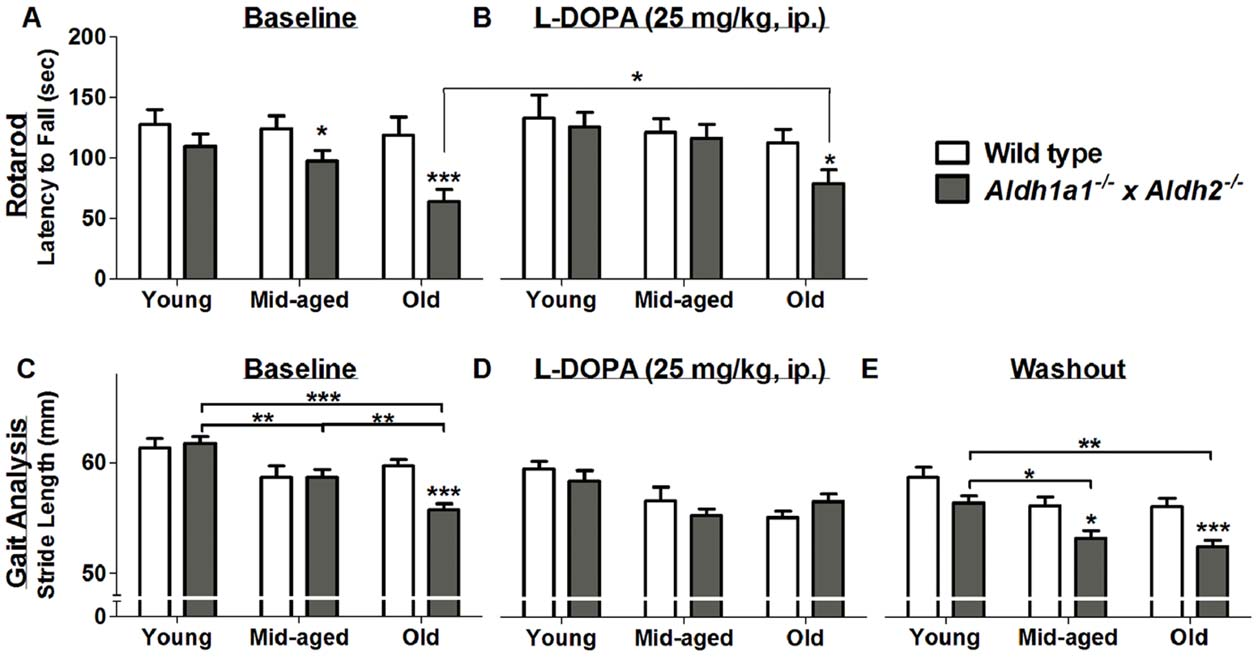
L-DOPA alleviated age-related motor deficits in *Aldh1a1^−/−^×Aldh2^−/−^* mice. Performance on the accelerating rotarod was measured in *Aldh1a1* ^−/−^×*Aldh2* ^−/−^ double knockout mice and age-matched wild type controls (A) before and (B) after L-DOPA treatment. Stride length was measured in *Aldh1a1* ^−/−^×*Aldh2* ^−/−^ double knockout mice and age-matched wild type controls (C) before and (D) after L-DOPA treatment. Measurement of stride length after elimination of L-DOPA (E) was performed 7 days after L-DOPA injection. Data in all panels are expressed as mean ± SEM of the following number of animals in each age group: young (wt = 5, ko = 6), middle-aged (wt = 5, ko = 13) and old (wt = 13, ko = 16) male mice. Asterisks above bars indicate significant difference between age-matched wt and ko groups. Asterisks above lines indicate significant difference between indicated means. *P<0.05; **P<0.01; ***P<0.001.

The effect of L-DOPA plus benserazide treatment on rotarod performance is shown in Figures 4A and 4B. There was a significant interaction between genotype and age (F_1,60_ = 6.73112, *p* = 0.0119) as well as between genotype and treatment (F_1,61_ = 9.74989, *p* = 0.0027) indicating that the change in rotarod performance with age and in response to treatment differs significantly depending on genotype. Data from wild type and *Aldh1a1^−/−^×Aldh2^−/−^* animals were analyzed separately. There was no significant effect of either age or L-DOPA in wild type animals but in *Aldh1a1^−/−^×Aldh2^−/−^* animals there was a significant decline in rotarod performance with age (F_1,32_ = 8.03070, *p* = 0.0079) and a significant improvement in response to L-DOPA (F_1,32_ = 17.69678, *p* = 0.0002). The age-treatment interaction was not significant (F_1,32_ = 0.55346, *p* = 0.4623), indicating that the response to L-DOPA was not dependent on age. After correcting for the effect of age, the *Aldh1a1^−/−^×Aldh2^−/−^* group had a significantly shorter mean latency to fall both before (F_1,60_ = 26.10433, *p*<0.0001) and after (F_1,60_ = 5.31982, *p* = 0.0246) L-DOPA treatment.

The effect of L-DOPA plus benserazide on gait analysis is shown in Figures 4C–E. Automated treadmill gait analysis revealed a significant interaction between genotype and treatment (F_1,60_ = 8.2298, *p* = 0.0057) on stride length, indicating that response to treatment differs significantly depending on genotype. Data from wild type and *Aldh1a1^−/−^×Aldh2^−/−^* animals were analyzed separately. There was a significant decline in stride length with age in wild type animals (F_1,28_ = 8.5845, *p* = 0.0067) and no other significant effects. After correcting for the effect of age, the *Aldh1a1^−/−^×Aldh2^−/−^* animals had significantly shorter rotarod times (F_1,60_ = 26.10433, *p*<0.0001) than wild type without L-DOPA and when treated with L-DOPA the difference was no longer significant (F_1,59_ = 1.996, *p* = 0.163). Parenthetically, the *Aldh1a1^−/−^×Aldh2^−/−^* animals also had a significantly shorter stride before L-DOPA treatment (F_1,59_ = 7.9885, *p* = 0.0064) but those measurements were not compared with L-DOPA due to the confounding effect of adaptation described in the methods.

### Effect of Genotype on Cognition

**Figure 5:**
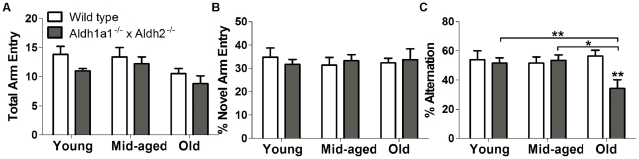
Effect of age and genotype on Y maze performance. (A) Total arm entries, (B) novel arm entries and (C) percent of arm alternations were measured in *Aldh1a1* ^−/−^×*Aldh2* ^−/−^ double knockout mice and age-matched wild type controls. Data are expressed as mean ± SEM of young (wt = 5, ko = 7), middle-aged (wt = 5, ko = 13) and old (wt = 13, ko = 13) male mice. Asterisks above bars indicate significant difference between age-matched wt and ko groups. Asterisks above lines indicate significant difference between indicated means. *P<0.05; **P<0.01; ***P<0.001.

The Y-maze test is a measure of hippocampal-dependent short-term memory. It is based on the innate preference of animals to remember and explore an arm that has not been previously explored. We recorded the percentages of novel arm entries and spontaneous alternations. As shown in Figure 5A, there was no significant effect of age (F_2,44_ = 2.768, df = 2, *p* = 0.074) or genotype (F_1,44_ = 2.013, *p* = 0.163) on total arm entry number. Figure 5B shows that there was no significant effect of age (F_2,44_ = 0.387, *p* = 0.681) or genotype on novel arm entries (F_1,44_ = 0.182, df = 1, *p* = 0.672). However, as shown in Figure 5C, there was a significant effect of genotype (F_1,44_ = 2.463, df = 1, *p* = 0.002) on the percent of alternation in the old *Aldh1a1^−/−^×Aldh2^−/−^* mice, but there was no significant effect of age (F_2,44_ = 1.338, *p* = 0.273). We found a significant interaction of age and genotype (F_2,44_ = 3.159, *p* = 0.05), so it is possible that there are age-dependent differences in percent alteration among wild type and *Aldh*-deficient animals if the genotypes were to be measured individually. By using one-way ANOVA followed by Student-Newman-Keuls *post-hoc* comparison, we found significant age-related differences (F_2,25_ = 4.230, *p* = 0.026) in old *Aldh1a1^−/−^×Aldh2^−/−^* mice compared to middle-aged (*p* = 0.026) and young (*p* = 0.048) mice. The percent alternation was not different in different age groups of wild-type mice (F_2,19_ = 0.244, *p* = 0.786). *Post-hoc* comparison of individual group means revealed that the oldest *Aldh1a1^−/−^×Aldh2^−/−^* mice had a significant decrease in the mean percentage of alternations as compared to wild-type controls (Figure 5C), indicating decreased working memory in the old *Aldh1a1^−/−^×Aldh2^−/−^* mice as compared to wild-type control.

### Effect of Genotype on Neostriatal Monoamines and Metabolites

**Figure 6:**
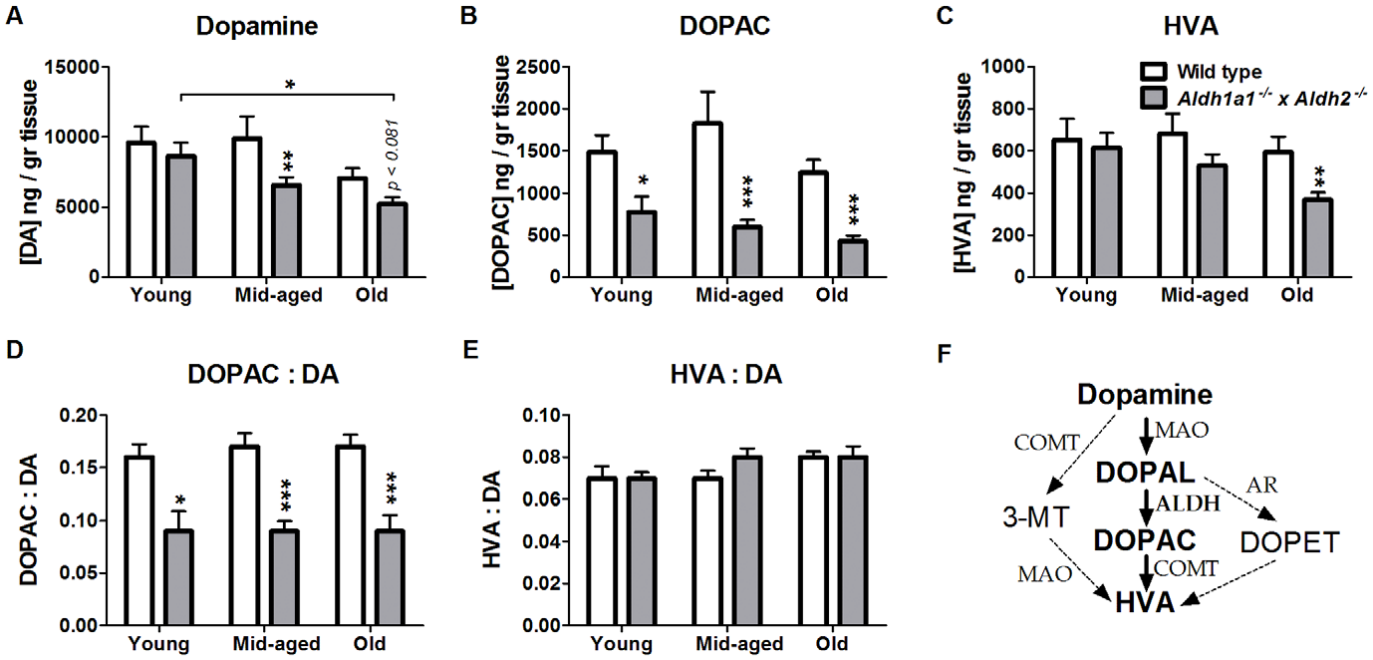
Effect of age and genotype on striatal dopamine and metabolites. Dopamine (A), DOPAC (B), HVA (C), DOPAC to DA ratio (D) and HVA to DA ratio (E) were measured in the neostriatum of *Aldh1a1* ^−/−^×*Aldh2* ^−/−^ double knockout mice and age-matched wild type controls. Pathways by which dopamine is metabolized to its metabolites are shown in (F). Data are expressed as the mean ± SEM of young (wt = 13, ko = 11), middle-aged (wt = 16, ko = 20) and old (wt = 23, ko = 32) male mice. Asterisks above bars indicate significant difference between age-matched wt and ko groups. Asterisks above lines indicate significant difference between indicated means. *P<0.05; **P<0.01; ***P<0.001.

Figure 6 shows the effects of age and genotype on dopamine and metabolites. As shown in Figure 6A, there was a significant effect of age (F_2,110_ = 5.996, *p* = 0.003) and genotype (F_1,110_ = 7.304, *p* = 0.008) on dopamine content. There was no statistically significant interaction between genotype and age (F_2,110_ = 0.778, *p* = 0.462). *Post-hoc* comparison of individual group means showedasignificant (*p* = 0.009) difference in dopamine between *Aldh1a1^−/−^×Aldh2^−/−^* mice and wild-type controls at middle age, and a marginally significant decrease (*p* = 0.081) in the oldest group. As shown in Figure 6B, there was a significant effect of genotype (F_1,110_ = 29.991, *p* = 0.001) but not age (F_2,110_ = 2.488, *p* = 0.088) on neostriatal DOPAC content. The difference in the mean values among the different age groups was not great enough to exclude the possibility that the difference is due to random sampling variability after allowing for the effects of differences in genotype. The interaction of age and genotype (F_2,110_ = 0.895, *p* = 0.412) was not significant. *Post-hoc* comparison of individual means revealed significant differences in mean levels of DOPAC between *Aldh1a1^−/−^×Aldh2^−/−^* and wild-type mice at all ages (Figure 6B). As shown in Figure 6C, there was a significant effect of genotype (F_1,110_ = 5.458, *p* = 0.021) and a marginally significant effect of age (F_2,110_ = 2.975, *p* = 0.055) on HVA content. There was no statistically significant interaction between genotype and age (F_2,110_ = 0.794, *p* = 0.454). *Post-hoc* comparison of individual group mean values for HVA content revealed a significant difference between *Aldh1a1^−/−^×Aldh2^−/−^* mice and the wild-type controls only in the oldest group.

**Figure 7:**
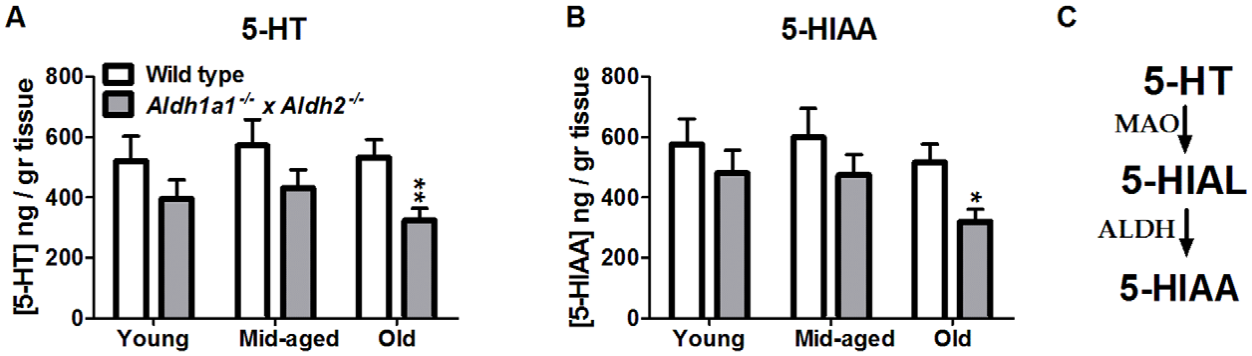
Effect of age and genotype on striatal 5-HT and 5-HIAA. The level of serotonin (A) and its metabolite, 5-HIAA (B) were measured in *Aldh1a1* ^−/−^×*Aldh2* ^−/−^ mice and age-matched wild type controls. Pathways by which 5HT is metabolized to its metabolites are shown in (C). Data are expressed as the mean ± SEM of the following number of wild-type and knockout mice in each age group: young (wt = 13, ko = 11), middle-aged (wt = 16, ko = 20) and old (wt = 23, ko = 32) male mice. *P<0.05, significantly different from age-matched wild-type control group. **P<0.01, significantly different from age-matched wild-type control group.

Figure 7 shows the effects of age and genotype on serotonin (5-hydroxytryptamine, 5-HT) and its metabolite, 5-hyrdoxy-indole acetic acid (5-HIAA). Figure 7A shows that there was a significant effect of genotype (F_1,110_ = 8.723, *p* = 0.004) on 5-HT content, but no significant effect of age (F_2,110_ = 0.825, *p* = 0.441). There was no interaction between age and genotype (F_2,110_ = 0.276, *p* = 0.76). *Post-hoc* analysis revealed a significant reduction in mean 5-HT content in *Aldh1a1^−/−^×Aldh2^−/−^* mice in the oldest group as compared to age-matched wild-type mice (*p* = 0.006). Figure 7B shows that there was a significant effect of age (F_2,110_ = 2.234, *p* = 0.112) and genotype (F_1,110_ = 5.817, *p* = 0.018) on 5-HIAA content. There was no statistically significant interaction between genotype and age (F_2,110_ = 0.3, *p* = 0.741). *Post-hoc* comparison of individual means revealed a significant difference between *Aldh1a1^−/−^×Aldh2^−/−^* mice and the wild-type controls in only the oldest group (*p* = 0.015).

### Effect of Genotype and Age on Aldehyde accumulation

**Figure 8:**
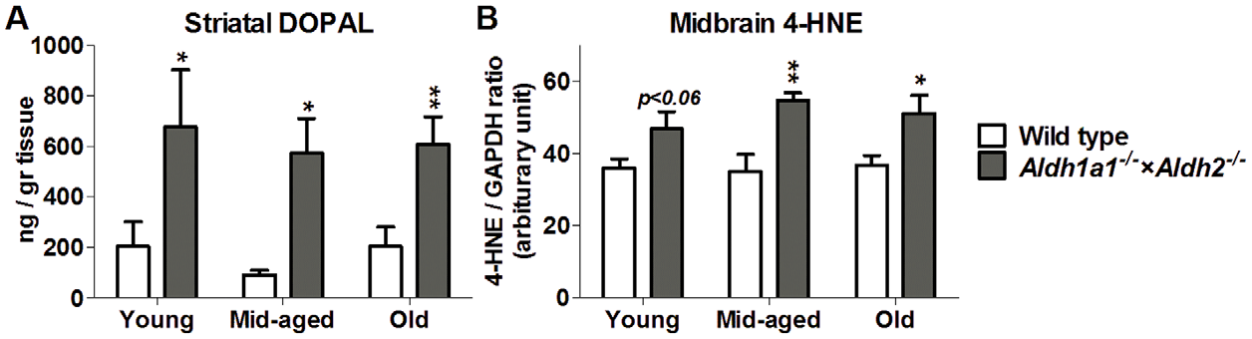
Effect of age and genotype on aldehyde accumulation in the nigrostriatal pathway. (A) Striatal DOPAL measured in the neostriatum of *Aldh1a1^−/−^×Aldh2^−/−^* double knockout mice and age-matched wild type controls. Data are expressed as mean ± SEM of young (wt = 5, ko = 7), middle-aged (wt = 5, ko = 9) and old (wt = 10, ko = 14) male mice. (B) Measurement of 4-HNE-protein adducts in midbrain of *Aldh1a1^−/−^×Aldh2^−/−^* double knockout mice and age-matched wild type controls. n = 4 per group. Data expressed as mean ± SEM. *P<0.05, significantly different from age-matched wild-type control group. **P<0.01, significantly different from age-matched wild-type control group.

Figure 8 shows striatal content of DOPAL (Figure 8A) and midbrain 4-HNE-protein adducts (Figures 8B and S2). Striatal DOPAL was measured by HPLC with electrochemical detection after batch alumina extraction. A subset of striatal tissue taken from the opposite side of the brain from those used to measure monoamines and metabolites was used for DOPAL measurement. A significant effect of genotype (F_1,44_ = 17.243, p<0.001) on DOPAL level was found; striatal DOPAL was elevated across ages of the *Aldh1a1^−/−^×Aldh2^−/−^* mice. There was no effect of age (F_2,44_ = 0.272, p = 0.763) on striatal DOPAL content and no significant interaction between age and genotype (F_2,44_ = 0.007, p = 0.993).

Figure 8B shows that the content of 4-HNE adducted proteins was elevated in middle age and old *Aldh1a1^−/−^×Aldh2^−/−^* mice. By semi-quantification of western blotting (Figure S2), there was significant effect of genotype (F_1,18_ = 22.874, p<0.001) on the intensity of 4-HNE-protein adducts in the midbrains of middle age (p = 0.002) and old (p = 0.017) *Aldh1a1^−/−^×Aldh2^−/−^* mice. No significant effect of age (F_2,18_ = 0.427, p = 0.659) nor interaction between age and genotype (F_2,18_ = 0.678, p = 0.520) were found on 4-HNE-protein adducts.

### Effect of Genotype and Age on the Number of Tyrosine Hydroxylase (TH) Immunoreactive Neurons in the Substantia Nigra Pars Compacta (SNpc)

**Figure 9:**
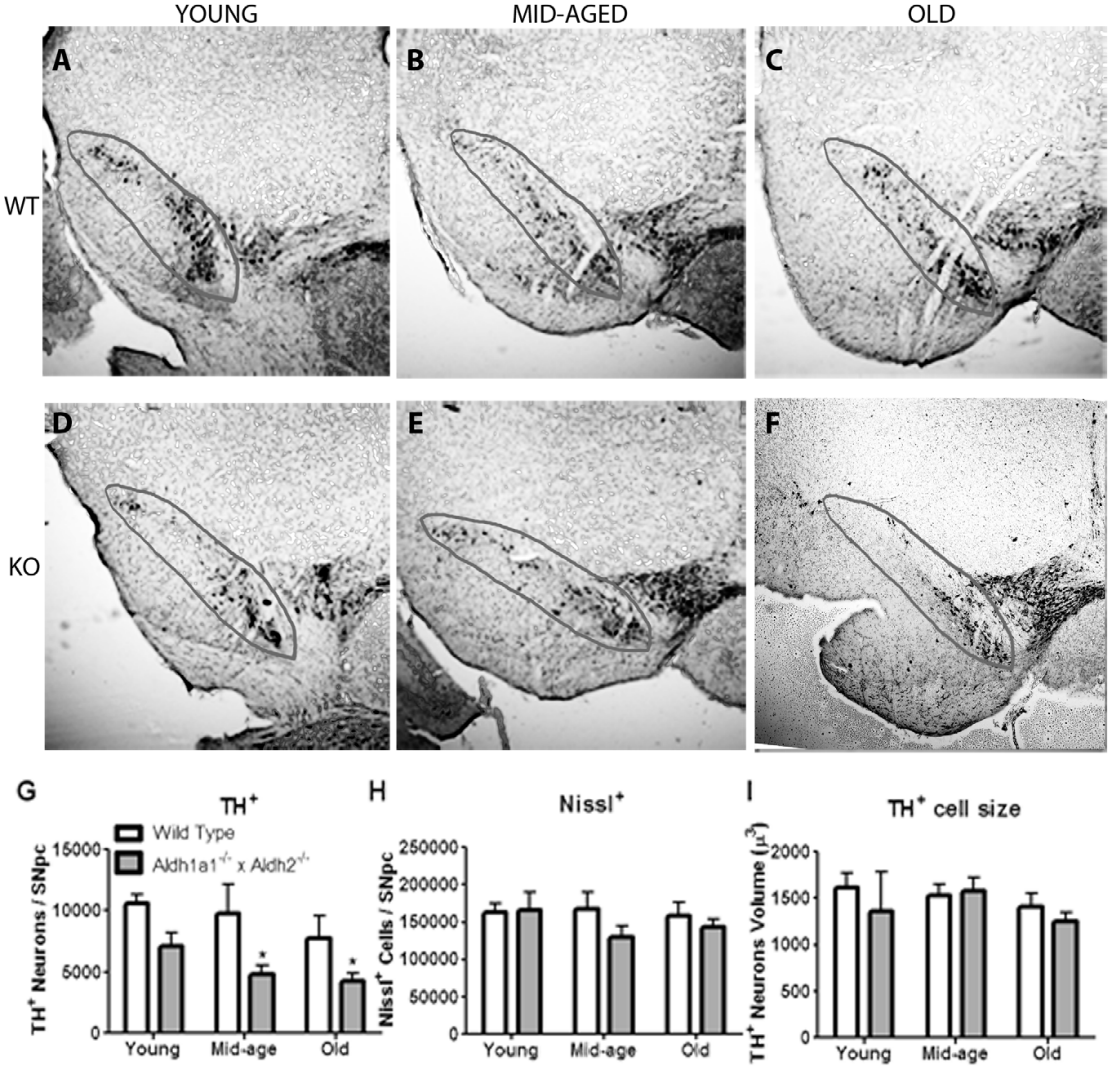
Effect of age and genotype on the number of dopamine neurons in the substantia nigra. (A–F) Representative coronal sections from *Aldh1a1* ^−/−^×*Aldh2* ^−/−^ mice and age-matched wild type controls showing regions of substantia nigra pars compacta (SNpc) selected for TH+ cell counts. Quantification data of (G) TH+ neurons, (H) Nissl cells, and (I) TH+ cell volume are expressed as the mean ± SEM of the following number of mice in each group: young (wt = 4, ko = 3), middle-aged (wt = 5, ko = 5) and old (wt = 5, ko = 9) male mice. *P<0.05, significantly different from age-matched wild-type control group.

Figure 9 shows the effects of age and genotype on cell morphometry in the midbrain. Unbiased stereology was used to estimate the number of dopaminergic neurons in the SNpc (Figure 9). As shown in Figure 9G, there was a significant effect of genotype (F_1,29_ = 13.054, *p*<0.001), but not age (F_2,29_ = 1.211, *p* = 0.313) on the number of TH-immunoreactive neurons. There was no statistically significant interaction between age and genotype (F_2,29_ = 0.089, *p* = 0.915). *Post-hoc* analysis of differences between individual means revealed significant 50% and 46% reductions in TH immunoreactive neurons in the middle-aged and old *Aldh1a1^−/−^×Aldh2^−/−^* mice, as compared to age-matched wild-type mice. Figure 9H shows that there was no significant effect of age (F_2,29_ = 0.610, *p* = 0.550) or genotype (F_1,29_ = 2.051, *p* = 0.163) on the number of Nissl-stained cells and no statistically significant interaction between age and genotype (F_2,29_ = 0.331, *p* = 0.721). Figure 9H shows that there was no effect of age (F_2,29_ = 0.708, *p* = 0.505) or genotype (F_1,29_ = 0.107, *p* = 0.748) on the size of TH-immunoreactive neurons and no significant interaction between age and genotype (F_2,29_ = 0.224, *p* = 0.802).

## Discussion

Aldehyde accumulation and/or impaired aldehyde detoxification has been hypothesized to play a role in the pathogenesis of PD [6], [19]–[22]. It was recently reported that patients who died with PD had an elevated DOPAL-to-dopamine ratio in caudate and putamen [11]. Aldehydes, including DOPAL and 4-HNE, are highly reactive and can modify proteins [6]–[9]. Aldehydes are also cytotoxic and have been demonstrated to kill dopaminergic cells *in vitro* and after intracranial injection [20], [21], [23]–[26]. A loss of function polymorphism in *ALDH2* has been reported to be a risk factor for Alzheimer's disease [16], [18], [27]. Others have reported that *ALDH1A1* mRNA is reduced in substantia nigra dopaminergic neurons in brains of PD patients [15] and *ALDH1A1* expression level in peripheral tissues has been reported as a candidate biomarker for PD diagnosis [28]. These findings are strongly suggestive of a role for reduced aldehyde detoxification in the development of neurodegenerative diseases. However, until now, direct evidence for a causal relationship between impaired aldehyde detoxification and neurodegeneration in PD has been lacking. We hypothesized that chronically decreased function of multiple aldehyde dehydrogenases consequent to impaired complex I, and/or reduced *ALDH* expression, plays a role in the pathophysiology of PD. To test this hypothesis, we generated a mouse model null for both *Aldh1a1* and *Aldh2*, the two isoforms of aldehyde dehydrogenase that are known to be expressed in midbrain dopaminergic neurons [13]–[15]. The results show that mutations in these two aldehyde dehydrogenase isoforms resulted in neurochemical evidence for ALDH inhibition and elevated striatal tissue DOPAL, followed by aging-related development of a PD-like phenotype.

Mice deficient in *Aldh1a1* and *Aldh2* exhibited age-related deficits in tests of motor performance that require a high degree of motor coordination. We found progressive age-related impairment in rotarod performance in the *Aldh1a1^−/−^×Aldh2^−/−^* mice as compared to wild-type controls. These effects were not due to alterations in muscle strength, body weight, motor learning, visual impairment or changes in circadian patterns of activity, factors that may confound the interpretation of rotarod performance [29]. This impairment in rotarod performance is consistent with what others have observed in animal models of PD. Rotarod performance has been shown to be impaired in genetic [30], [31] and toxin-induced [32] animal models of PD.

Altered gait pattern is symptomatic of PD [33]–[35]. A behavioral hallmark of PD is a shortened stride length, which is manifested as a shuffling gait [34], [35]. Alterations in gait have also been observed in animal models of PD and in models of other basal ganglia diseases, including Huntington's disease [36]–[38]. Conversely, others have reported that gait analysis did not detect alterations in the MPTP model of PD or in a model of amyotrophic lateral sclerosis [39]. In the present study, gait analysis revealed asignificant 8.3% reduction of stride length in the oldest *Aldh1a1^−/−^×Aldh2^−/−^* mice. A similar magnitude of reduction in stride-length was reported for mice treated with MPTP [40]. That paper also reported a similar shorter stride duration and stride frequency [40]. We found no differences in stride duration and frequency. On the other hand, others have reported no differences in stride length, but shorter stride duration and frequency in mice overexpressing human α-synuclein [41]. Unlike MPTP-treated mice, those mice showed no loss of dopamine neurons. Thus, gait parameters may be differentially affected by the underlying pathology.

Responsiveness to L-DOPA treatment is diagnostic of PD [42], [43]. To test whether *Aldh1a1^−/−^×Aldh2^−/−^* mice were responsive to L-DOPA treatment, we treated mice with L-DOPA plus benserazide, a peripheral aromatic amino acid decarboxylase inhibitor. The dose that we used (25 mg/kg body weight) was chosen based on calculations of the human equivalent dose (∼2.03 mg/kg) as reported by FDA Draft Guidelines [44], [45]. This dosage is also one that is commonly reported for mice in the literature [46], [47]. L-DOPA treatment did not alter rotarod or gait performance of wild-type mice as compared to the baseline measurements. However, L-DOPA treatment alleviated the age-related deficits in stride length and rotarod performance in *Aldh1a1/Aldh2*-deficient mice. Furthermore, deficits in stride length reappeared a week after L-DOPA treatment, a time when L-DOPA had been completely eliminated. Thus, with respect to responsiveness to L-DOPA, the *Aldh1a1/Aldh2*-deficient mice model this aspect of Parkinson's disease. One might argue that L-DOPA administration would enhance aldehyde accumulation and toxicity and thus impair performance in *Aldh1a1/Aldh2*-deficient mice. However, we feel that it is unlikely that a single L-DOPA injection would cause immediate and measureable cytotoxicity and/or augmentation of dopaminergic neurodegeneration.

Cognitive impairment is often found in PD [48]. For example, patients with mild, early stage, Parkinson's disease are impaired in planning and spatial working memory tests [49]. Therefore, we measured cognitive function by a Y-maze test, an assessment of short-term spatial working memory that is based on the innate tendency of rodents to explore novel environments. The advantage of the Y-maze test is that it is not as dependent on motor function as compared to other tests of memory, such as the Morris water maze [50]. The Y-maze paradigm is also less stressful and less influenced by emotionality [51]. In the present study, there were no differences due to genotype or age in total or novel arm entries, measures of locomotion and exploratory behavior respectively. However, there was a significant effect of age and genotype on the percentage of arm alternations, a measure ofspatial working memory. Thus, at the oldest age, the *Aldh1a1^−/−^×Aldh2^−/−^* mice were significantly impaired as compared to wild-type mice. Deficiency in short-term spatial working memory, as measured in a T-maze, has been previously reported in mice treated with moderate doses of MPTP to mimic early stage PD [52]. In that study, the dose used produced a 50 to 60% decrease in dopamine, but had no effect on locomotor activity in an open field test, similar to the levels of impairment in these parameters that we observed in our model in the present study. Similar effects of low dose MPTP on spatial working memory have been reported in monkeys [53]. Thus, the effects of age and *Aldh1a1*/*Aldh2*-deficiency on spatial memory recapitulate the cognitive deficits seen in early stage Parkinson's disease.

We also measured monoamines and metabolites in the neostriatum. The serotonergic system has been reported to be affected in PD patients, although to a smaller extent than the dopaminergic system [54]. Reductions in serotonin and its metabolite 5-HIAA in caudate nucleus and putamen of PD patients have been reported [55], [56]. Several studies have linked complications found in PD, such as fatigue, depression and dementia, to dysfunction of the serotonergic system [1], [57], [58]. Consistent with these findings, the *Aldh1a1^−/−^×Aldh2^−/−^* double knockout mice exhibited decreased striatal 5-HT (by 39%) and its metabolite 5-HIAA (by 38%), only at the oldest age.

Deficiency in *Aldh1a1* and *Aldh2* was also associated with significant decreases in dopamine and its metabolites DOPAC and HVA in middle aged and old mice. The deficits in dopamine and HVA content were only significant in the middle-aged and oldest group, respectively. In contrast, DOPAC was reduced by 48 to 67% of control at all ages and DOPAL was increased at all ages. The deficiency in DOPAC and the increase in DOPAL is consistent with the idea that Aldh1a1 and/or Aldh2 play a role in metabolism of DOPAL to DOPAC. We previously reported that deficiency in *Aldh2* was not associated with alterations in dopamine or metabolites [59]. Recently, it was reported that in *Aldh1a1* deficient mice 2–3 months of age, there was no reduction in striatal dopamine levels and that DOPAC was slightly but significantly reduced by 20% as compared to wild-type control mice [60]. The up to 67% reduction in DOPAC in the present study, as compared to the much smaller reduction in the *Aldh1a1*-deficient mice reported previously [60], supports the idea that more than one aldehyde dehydrogenase participates in metabolizing DOPAL to DOPAC. A recent clinical study reported that DOPAL-to-dopamine ratio was significantly elevated in the caudate and putamen of PD patients [11]. The reduced ability to detoxify DOPAL by metabolizing it to DOPAC may contribute to the degeneration of dopamine neurons in PD. Thus, in the present study, elevated striatal DOPAL preceded evidence of loss of dopaminergic terminals.

In addition to the elevated DOPAL content, we observed increases in 4-HNE adducted proteins in the midbrain. Together, these results provide direct evidence that Aldh1a1 and Aldh2 are necessary to detoxify multiple biogenic aldehydes. Since each of these is known to be cytotoxic, this suggests that accumulation of multiple biogenic aldehydes may contribute to the neurodegeneration observed in PD. Thus, DOPAL and 4-HNE have been reported to induce death of cultured cells [22], [61]. Moreover, DOPAL has been reported to cause degeneration of dopaminergic neurons when injected intracranially [20]. In addition, DOPAL and 4-HNE have been reported to accelerate oligomerization of α-synuclein and such oligomers may also be cytotoxic [8], [9].

A loss of TH-immunoreactive cells was observed in *Aldh1a1^−/−^×Aldh2^−/−^* mice. A statistically significant reduction of up to 50% was observed in middle-aged and old *Aldh1a1^−/−^×Aldh2^−/−^* mice as compared to their age-matched wild-type controls. The loss of TH-immunoreactive neurons is consistent with the loss of striatal dopamine. However, the loss of striatal dopamine was smaller in magnitude than the reduction in TH-immunoreactive neurons. It has long been reported that in studies of post-mortem human brain tissue, the degree of loss of substantia nigra dopamine neurons is greater than the loss of dopamine and metabolites in striatal regions, suggesting that surviving neurons have an increased capacity to make and store dopamine [62]. There was no significant difference in the number of Nissl-stained cells, indicating that the reduction in TH^+^ neurons was relatively specific. There was also no difference in the size of the TH^+^ cells. There is no preclinical or clinical evidence showing a relationship between *Aldh2* deficiency and neurochemical and behavioral manifestations of PD. However, the loss of TH^+^ cells in the *Aldh1a1^−/−^×Aldh2^−/−^* mice is in contrast to a recent report that TH^+^ neurons are increased in *Aldh1a1*-deficient mice [60]. Taken together, these findings suggest that deficiency in only one aldehyde dehydrogenase isoform is insufficient to cause neuropathology and that multiple redundant mechanisms for aldehyde detoxification participate in protecting dopamine neurons from the toxic effects of aldehydes.

In summary, deletion of two isoforms of aldehyde dehydrogenase, *Aldh1a1* and *Aldh2*, which are known to be present in dopamine neurons, resulted in a Parkinsonian phenotype characterized by age-dependent deficits in motor performance, age-related reductions in monoamines and their metabolites and loss of neurons in the substantia nigra. These results provide the first direct support for the hypothesis that impaired aldehyde detoxification plays a role in PD. The *Aldh1a1^−/−^×Aldh2^−/−^* mouse may be a useful new model of early stage Parkinson's disease.

## Materials and Methods

### Animals and Ethics Statement

Animals were maintained, and experiments were conducted, in strict accordance with the *Institutional Animal Care and Use Committee (IACUC), The University of Texas Health Science Center at San Antonio* and *the South Texas Veterans Health Care System* (San Antonio, TX), and with the *1996 Guide for the Care and Use of Laboratory Animals (Institute of Laboratory Animal Resources on Life Sciences, National Research Council, National Academy of Sciences)*. All protocols were designed to minimize animal discomfort. Animals were group housed with five per cage in ventilated cages that were maintained at an ambient temperature of 23–25°C and on a 12-hour light-dark cycle. Rodent bedding consisted of Sani-Chips (Harlan Teklad, Madison, WI). Mice had free access to food (Teklad 7912, Rodent sterilized diet, Harlan Teklad, Madison, WI) and water. The *Aldh1a1^−/−^×Aldh2^−/−^* mice used in this study were generated by crossing mice with a targeted mutation in *Aldh1a1* with a line of mice carrying a mutation in *Aldh2*. Mice null for *Aldh2* were generated by gene trap mutagenesis as we have previously reported [59] and were backcrossed to C57BL/6J mice for 10 generations. The *Aldh1a1* mutant mice were generated by Gregg Duester and colleagues using a targeted deletion at exon 11 of the *Aldh1a1* allele [63] and were backcrossed by us for 8 generations to C57BL/6J. *Aldh1a1^−/−^* mice were crossed with *Aldh2^−/−^* mice to produce mice heterozygous for both genes, *Aldh1a1^+/−^×Aldh2^+/−^*. Cross-breeding of *Aldh1a1^+/−^×Aldh2^+/−^* generated the *Aldh1a1^−/−^×Aldh2^−/−^* line and the wild type *Aldh1a1^+/+^×Aldh2^+/+^* line. The two lines were maintained by breeding male and female *Aldh1a1^−/−^×Aldh2^−/−^* or *Aldh1a1^+/+^×Aldh2^+/+^* mice. Male mice of three different age groups (young, 5–8 months; middle-aged, 12–14 months; and old, 18–27 months), were used for the study. Body weights were monitored prior to and during behavioral testing. Treatments and behavioral tests were blinded, randomly assigned, and performed during the light cycle from 8am to 6pm if not otherwise indicated.

### Grip Strength

Mice were allowed to grip the metal grids of a grip meter (Ametek Chatillon) with their forepaws, and they were gently pulled backwards by the tail until they could no longer hold the grids. The peak grip strength observed in 5 trials was recorded.

### Open Field Activity and 24-hour Locomotor Activity

For open field activity, mice were placed in an open field arena (40×40×40 cm); for 24-hour locomotor recording, mice were placed in plastic boxes (48×24×20 cm) and interruptions of the photo-beams were recorded and analyzed using compatible computer software. Data were collected for 30 min and assessed at five-min intervals for locomotor activity. For 24-hour activity, each test session started at 5 pm and ended at 6 pm the next day with food and water available *ad libitum*. Data were collected at one-hour intervals.

### Accelerating Rotarod Performance

Mice were trained on the rotarod for two days and then tested on the third day. Three trials were performed on each day. During each trial, the rotarod began at an initial speed of 4 rpm and then increased gradually to 40 rpm within 300 seconds. The averaged latency to fall from the accelerating rod was calculated from the three trials performed on the third day.

### Automated Gait Analysis

Animals were tested on the ExerGait (Columbus Instruments) treadmill at a speed of 15 cm/sec for 10 sec. A digital camera recorded the reflected images of the footpads. The digital camera was operated at 80 frames per second and the output was fed directly to computer. Video recordings were analyzed by TreadScan™ 1.0 software (Clever Sys Inc.). The average of each parameter was collected from the strides within a 20–100 mm range.

### Y-maze Test

To measure the effect of genotype on cognitive performance, mice were tested in a three-armed Y-maze test. Mice were pre-trained with one arm blocked (i.e., arm C) and allowed to move freely in the other two arms. The percentage of the triads in which all three arms were represented (ABC, CAB, or BCA but not BAB) were recorded on the 5-min testing trial.

### L-DOPA Drug Administration

After baseline measures of gait and rotarod performance, animals received 25 mg/kg L-DOPA (Sigma) administered concurrently with 12.5 mg/kg benserazide intraperitoneally. Motor function was assessed within the 30–120 min peak therapeutic window for L-DOPA.

### Preparation of Brain Tissue

Brains were rapidly collected by decapitation following brief CO_2_ inhalation. For neurochemical assays, brain tissue was rapidly dissected on an ice-cold platform; tissue was then snap-frozen on dry ice and transferred to a −80°C freezer for storage. Samples were homogenized in ice-cold 0.1 M perchloric acid (HClO_4_) for HPLC analysis or in NP-40 lysis buffer (Fluka) for Western blotting protein extraction. Brain samples for immunohistochemical analyses were removed, subsequently stored overnight in 4% paraformaldehyde (PFA; Affymetrix) at 4°C and then cryoprotected in 30% sucrose. A cryostat was used to cut serial 40 µm coronal sections through the midbrain and then stored in a cryopreservative buffer consisting of 30% glycerol/30% ethylene glycol in 100 mM sodium phosphate at −20°C.

### Measurement of Monoamine and Metabolites

Samples were homogenized in ice-cold 0.1 M HClO_4_ containing 10 ng/mL DHBA (3,4-dihydroxybenzylamine) as the internal standard and centrifuged to remove precipitated proteins. The supernatants were used for HPLC analysis. An aliquot of the supernatant was filtered through a 0.45-mm microcentrifuge filter (Millipore). Dopamine, serotonin, and metabolites were separated using a high-performance liquid chromatography (HPLC, ESA) system with an HR-80 reverse-phase C18 column (4.6×80 mm). Samples were detected with an electrochemical detector (ECD) on a dual-electrode analytical cell (Model 5011A). The pH of the 6% acetonitrile (v/v) mobile phase was adjusted to pH 3.11 with phosphoric acid after the addition of organic modifiers. The flow rate of mobile phase was set at 1.0 mL/min.

### Measurement of the aldehyde intermediate of dopamine metabolism, DOPAL

DOPAL was measured at the Catecholamine Resource Unit of the Clinical Neurocardiology Section of the NINDS, according to a previously described method [11]. Samples were homogenized in an acid solution containing 20 ∶ 80 parts of 0.2 M acetic acid and phosphoric acid solution. After homogenization, samples were centrifuged and the supernatant was frozen and stored at −80°C freezer for storage until assayed for DOPAL by HPLC with electrochemical detection after batch alumina extraction. Briefly, mobile phase containing octanesulfonic acid as an ion pairing agent was pumped isocratically through a reverse phase liquid chromatographic column. Catechols were quantified by the current produced upon exposure of the eluate to flow-through electrodes set to oxidizing and then reducing potentials in series, with recording from the last electrode reflecting reversibly oxidized species.

### Measurement of 4-HNE-protein adducts

Midbrains were homogenized in NP-40 lysis buffer (Fluka). Protein concentrations were determined using the Bradford assay (Bio-Rad, Hercules, CA, USA) following the manufacturer's instructions. Total protein of 40 µg per sample were subjected to 4–11% SDS-PAGE (Bio-Rad; sodium dodecyl sulfate-polyacrylamide gel electrophoresis). Separated proteins were then transferred onto a nitrocellulose membrane (0.45 µm, PROTRAN, Whatman) using 20% methanol wet transfer for immunostaining. The membrane was blocked for 1 hour in Odyssey blocking solution (LI-COR). Content of 4-HNE-protein adducts (R&D, 1∶1,000) was analyzed by an Odyssey IR scanner (LI-COR) with appropriate secondary IRDye (LI-COR) antibody.

### TH Immunohistochemistry

Free floating sections were washed with 1× phosphate-buffered saline (PBS), and endogenous peroxidases were quenched by 1% hydrogen peroxide for 10 minutes at room temperature (RT), followed by rinses with 1× PBS. After sections were blocked with 1% Bovine serum albumin, 3% normal goat serum and 0.3% TritonX-100, they were stained with rabbit anti-TH (1∶1000; Pel-Freeze) for 48 hours at 4°C. Following primary incubation, sections were washed with 1× PBS and incubated in biotinylated anti-rabbit secondary antibody (1∶ 400; Vector Laboratories) with blocking buffer for 60 minutes at RT. Afterward, sections were washed with 1× PBS and then re-incubated in ABC solution (Vectastain ABC kit; Vector Laboratories) for 30 minutes at RT. For color development, sections were treated with 3-diaminobenzidine (DAB) as the chromogen with nickel chloride amplification. Finally, sections were mounted on positively charged slides, counterstained with 0.1% cresyl violet (Nissl staining) and cover-slipped.

### Measurement of Midbrain Dopamine Neurons by Unbiased Stereology

Unbiased stereology methodology was used to estimate the number of tyrosine hydroxylase positive (TH^+^) and Nissl-stained (Nissl^+^) cells in the SNpc. Cells were visualized by a Nikon microscope, and images were relayed via a MicroFibre digital camera (http://www.optronics.com) to a computer where they were counted by a blinded observer using the optical fractionator the Stereologer system (Stereology Resource Center, Chester, MD). Every sixth section was collected through the SNpc beginning at bregma – 2.06 mm and finishing at bregma – 4.16 mm. Sections were cut at a thickness of 40 µm to allow an average of 18 µm thick optical dissector within each section after staining and dehydration processes. Reference spaces on each section were outlined under low magnification (4×), and TH^+^ and Nissl^+^ neurons were counted at high magnification (40×), with a guard volume of 1 µm on both surfaces to avoid artifacts on the cut surface of sections. The first sample section from the first 1–6 sections collected was chosen at random. TH^+^ cells were counted in grids randomly positioned by the software in the outlined counting area through all optical planes, thus creating a three-dimensional counting area. The counting criteria included the presence of TH immunoreactivity in the cytoplasm of cells with a neuronal phenotype, including a clear nuclear membrane and distinct nucleolus. We also used an additional stereological probe, the rotator method, to measure cell volume.

### Statistical Analysis

Significance of the effects of age and genotype was determined by two-way ANOVA with age and genotype as factors. Differences between individual means were assessed by the Student-Newman-Keuls *post-hoc* comparison to assess differences between individual means. For experiments involving multiple measures to analyze the effect of L-DOPA administration on gait and rotarod performance, a mixed-effect ANCOVA model with first order interactions was fitted to the data. Genotype and L-DOPA were used as categorical variables, age as a numeric variable, and different animals as a grouping variable. Likelihood ratio tests were used to determine which random effects to include. For rotarod, random-intercept and random-slope terms were included in the model and for gait, only a random-intercept term was necessary. When there were significant interactions involving genotype, data from the wild type and *Aldh*-deficient were analyzed separately using a reduced version of the above model. To determine the effect of genotype alone while taking into consideration the effect of age, a one-way mixed-effect ANOVA with random intercepts and age as a grouping variable was fitted separately to baseline and L-DOPA data. In the case of gait analysis, to correct for adaptation to the apparatus, performance after a one-week post L-DOPA washout period was used as the baseline measurement rather than the initial pre L-DOPA measurement. For the mixed-model analysis, the R statistical language [64] with the nlme package [65] were used.

## Supporting Information

Figure S1 **Effect of** ***Aldh1a1*** **^−/−^×** ***Aldh2*** **^−/−^ genotype on locomotor activity.** (A) General locomotor activity measured in an open field, (B) light/dark open field activity test and (C) 24-hour activity tests. Data are expressed as the mean ± SEM of the number of wild type and knockout mice in the following age-groups: young (wt = 5, ko = 7), mid-aged (wt = 11, ko = 12) and old (wt = 7, ko = 9) male mice.
(TIF)

Figure S2 **Effect of age and genotype on midbrain 4-HNE-protein adducts.** Figure S2 shows the western blot of 4-HNE-adducts in midbrains of *Aldh1a1* ^−/−^×*Aldh2* ^−/−^ mice and age-matched wild type controls. Integrated intensity was measured using Odyssey IR scanner (LI-COR). An integrated density ratio was calculated from the intensity of the whole lane of 4-HNE-adducted proteins divided by the intensity of the GAPDH band corrected for background. Background was obtained from the top and bottom of each lane.
(TIF)
