# Supplementary figures and images for: Neurodegeneration and Motor Dysfunction in Mice Lacking Cytosolic and Mitochondrial Aldehyde Dehydrogenases: Implications for Parkinson's Disease
Source: PLoS One. 2012 Feb 22;7(2):e31522. doi: 10.1371/journal.pone.0031522 (PMC3284575; doi:10.1371/journal.pone.0031522)

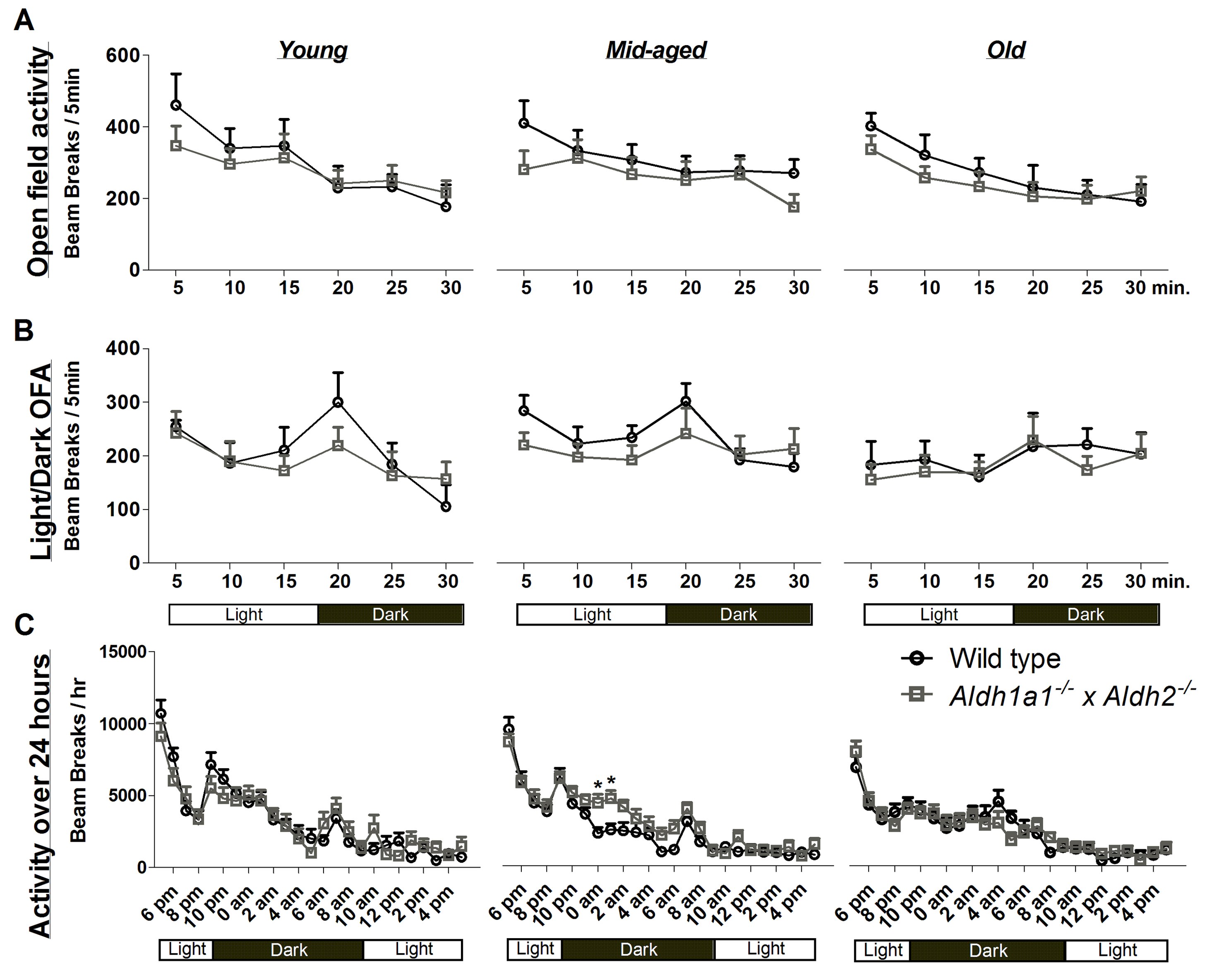

Supplement: Figure S1 — Effect of Aldh1a1 −/−× Aldh2 −/− genotype on locomotor activity. (A) General locomotor activity measured in an open field, (B) light/dark open field activity test and (C) 24-hour activity tests. Data are expressed as the mean ± SEM of the number of wild type and knockout mice in the following age-groups: young (wt = 5, ko = 7), mid-aged (wt = 11, ko = 12) and old (wt = 7, ko = 9) male mice. (TIF) [file pone.0031522.s001.tif]

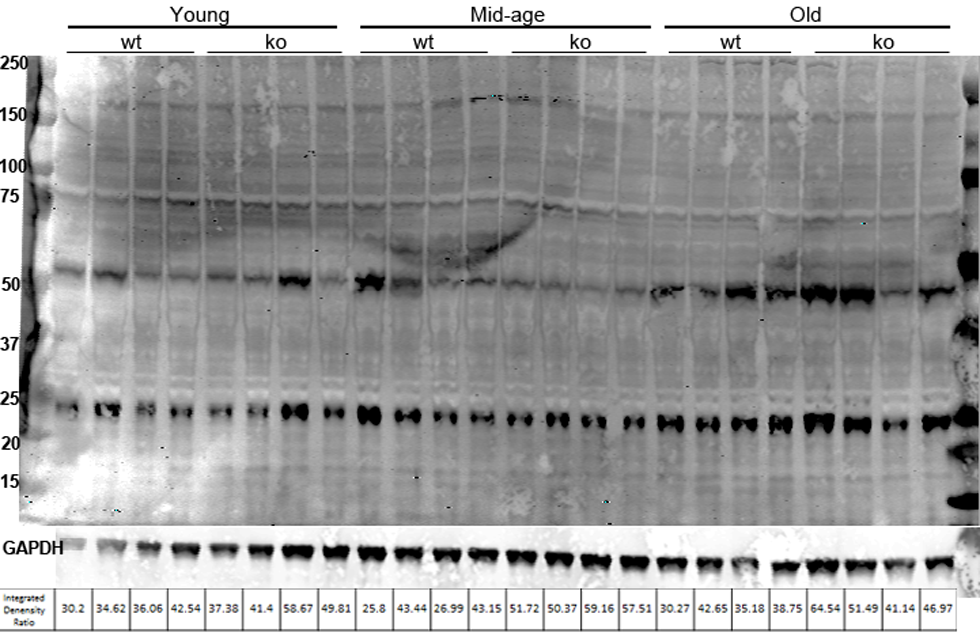

Supplement: Figure S2 — Effect of age and genotype on midbrain 4-HNE-protein adducts. Figure S2 shows the western blot of 4-HNE-adducts in midbrains of Aldh1a1 −/−×Aldh2 −/− mice and age-matched wild type controls. Integrated intensity was measured using Odyssey IR scanner (LI-COR). An integrated density ratio was calculated from the intensity of the whole lane of 4-HNE-adducted proteins divided by the intensity of the GAPDH band corrected for background. Background was obtained from the top and bottom of each lane. (TIF) [file pone.0031522.s002.tif]
